# Epigenetic Telomere Protection by *Drosophila* DNA Damage Response Pathways

**DOI:** 10.1371/journal.pgen.0020071

**Published:** 2006-05-19

**Authors:** Sarah R Oikemus, Joana Queiroz-Machado, KuanJu Lai, Nadine McGinnis, Claudio Sunkel, Michael H Brodsky

**Affiliations:** 1 Program in Gene Function and Expression and Program in Molecular Medicine, University of Massachusetts Medical School, Worcester, Massachusetts, United States of America; 2 Instituto de Biologia Molecular e Celular, Universidade do Porto, Porto, Portugal; 3 Faculdade de Ciências da Saúde, Universidade Fernando Pessoa, Porto, Portugal; 4 Instituto de Ciências Biomédicas de Abel Salazar, Universidade do Porto, Porto, Portugal; Stowers Institute for Medical Research, United States of America

## Abstract

Analysis of terminal deletion chromosomes indicates that a sequence-independent mechanism regulates protection of *Drosophila* telomeres. Mutations in *Drosophila* DNA damage response genes such as *atm/tefu, mre11,* or *rad50* disrupt telomere protection and localization of the telomere-associated proteins HP1 and HOAP, suggesting that recognition of chromosome ends contributes to telomere protection. However, the partial telomere protection phenotype of these mutations limits the ability to test if they act in the epigenetic telomere protection mechanism. We examined the roles of the Drosophila atm and *atr-atrip* DNA damage response pathways and the *nbs* homolog in DNA damage responses and telomere protection. As in other organisms, the *atm* and *atr-atrip* pathways act in parallel to promote telomere protection. Cells lacking both pathways exhibit severe defects in telomere protection and fail to localize the protection protein HOAP to telomeres. Drosophila nbs is required for both *atm-* and *atr*-dependent DNA damage responses and acts in these pathways during DNA repair. The telomere fusion phenotype of *nbs* is consistent with defects in each of these activities. Cells defective in both the *atm* and *atr* pathways were used to examine if DNA damage response pathways regulate telomere protection without affecting telomere specific sequences. In these cells, chromosome fusion sites retain telomere-specific sequences, demonstrating that loss of these sequences is not responsible for loss of protection. Furthermore, terminally deleted chromosomes also fuse in these cells, directly implicating DNA damage response pathways in the epigenetic protection of telomeres. We propose that recognition of chromosome ends and recruitment of HP1 and HOAP by DNA damage response proteins is essential for the epigenetic protection of *Drosophila* telomeres. Given the conserved roles of DNA damage response proteins in telomere function, related mechanisms may act at the telomeres of other organisms.

## Synopsis

Organisms with linear chromosomes must distinguish between the naturally occurring ends of chromosomes (telomeres) and chromosome breaks due to DNA damage. Many eukaryotic cells use DNA binding proteins that specifically recognize telomeric DNA sequences to protect telomeric DNA ends from the inappropriate action of DNA repair enzymes. In *Drosophila melanogaster,* however, chromosomes that lack telomere-specific sequences can be isolated and stably maintained. Thus, telomere protection can be inherited via a sequence-independent or epigenetic mechanism. Oikemus et al. demonstrate that two groups of genes that help cells respond to DNA damage are also required to localize a telomere protection protein to chromosome ends. Two experiments are described to support the model that these DNA damage detection genes help maintain telomere protection regardless of telomere sequence. First, mutations in these genes lead to loss of telomere protection without loss of telomeric DNA. Second, telomeres that lack all telomere-specific sequences still require these genes for protection. Combined, these experiments suggest that recognition of DNA ends is required for sequence-independent protection of Drosophila melanogaster telomeres. Since the same genes also promote telomere protection in yeast, plants, and mammals, these observations may be relevant to chromosome function in many organisms.

## Introduction

The ends of eukaryotic chromosomes can be protected from end-to-end fusion by two distinct mechanisms. In most organisms, sequence-specific DNA binding proteins recognize telomere-specific sequences and protect telomeres from the activity of DNA repair systems [1,2]. However, genetic studies in *Drosophila* have demonstrated that telomeres can also be protected from end-to-end fusion by an epigenetic mechanism. The telomeric DNA of *Drosophila* chromosomes is composed of retrotransposons and repetitive telomere-associated sequences [3]. Terminal deletion chromosomes that completely lack these sequences can be recovered and propagated [4–8]. The telomeres of these chromosomes are protected from fusion and do not induce DNA damage responses such as cell cycle arrest or apoptosis. These observations demonstrate that a sequence-independent mechanism can protect *Drosophila* chromosomes from telomere fusion and suggest that a similar mechanism contributes to protection of normal telomeres. The sequence-independent inheritance of telomere protection is conceptually similar to the epigenetic regulation of centromere function in which the function of a chromosomal domain is usually associated with a specific set of sequences, but can be stably transferred to alternative sequences [9,10]. Thus, *Drosophila* telomere protection can be grouped with centromere function and gene expression as processes that can be regulated by an epigenetic mechanism.

Two chromatin-associated proteins, HP1 and HOAP, are required for telomere protection and localize to the telomeres of both normal and terminally deleted chromosomes [11–13]. The role of HP1 in the epigenetic inheritance of chromatin modifications during cell division [14] suggests that a similar activity may contribute to telomere protection. Inheritance of chromatin modifications is often initiated or stabilized by specific chromosome features, such as binding sites for sequence-specific DNA binding proteins or repeat sequences at centromeres [15,16]. The stable inheritance of terminally deleted chromosomes over many generations indicates that a feature of telomeres other than telomere-specific sequences can recruit or maintain HP1 and HOAP at telomeres.

**Figure 1:**
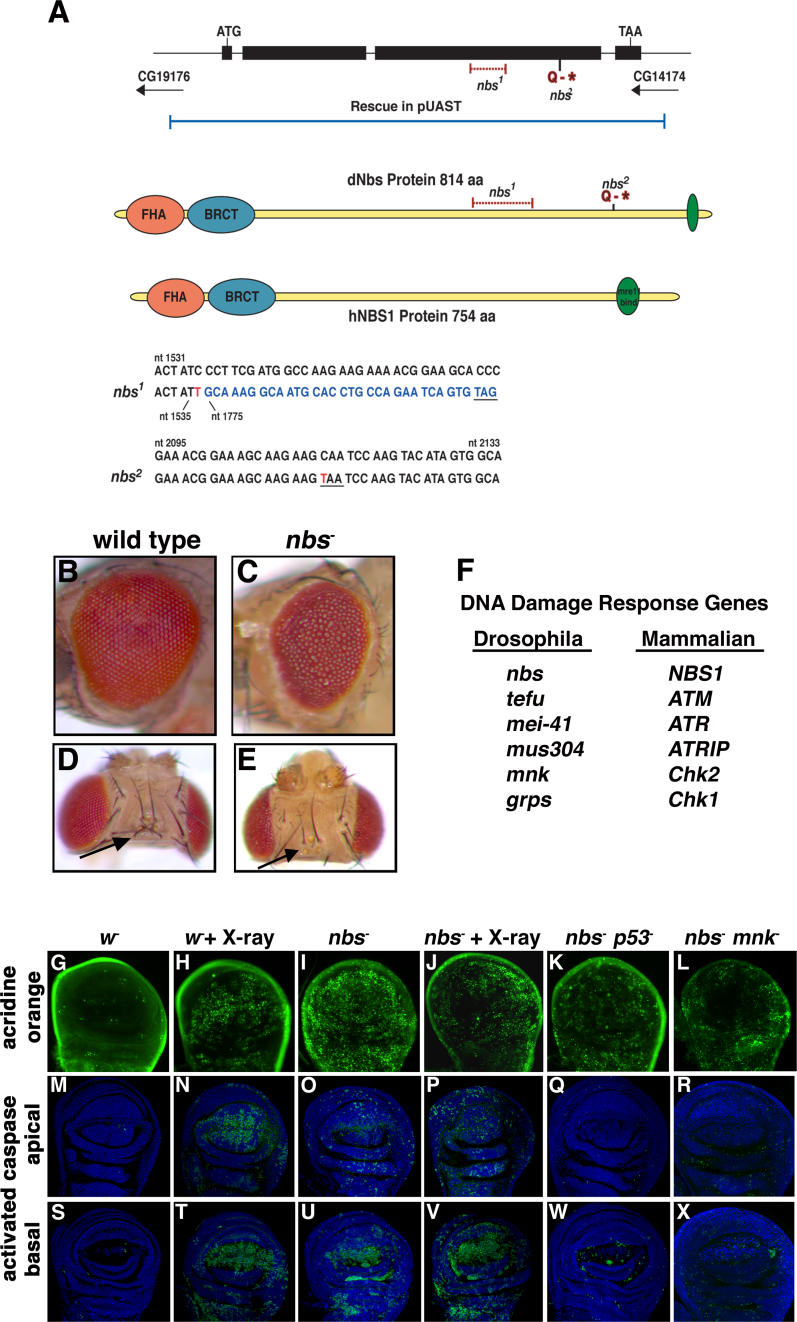
Drosophila nbs Is an Essential Gene Required for DNA Damage–Induced Apoptosis (A) Upper: *nbs* gene structure. The Drosophila nbs gene is composed of four exons and encodes a protein (dNbs) with similar domain structure to the human NBS1 protein (hNBS1). *nbs* mutations are indicated in red. The P[*nbs^+^*] genomic rescue construct is shown in blue. Middle: Nbs protein structure. *Drosophila* and human Nbs protein structures are depicted with the forkhead-associated (FHA) domain in orange, the BRCT domain in blue, and the Mre11-interacting domain in green. Lower: alignment of wild-type and mutant *nbs* genomic DNA sequences. The *nbs^1^* mutation is a 238-bp deletion and single bp insertion at nucleotide position 1,536 that results in a frameshift and a new stop codon (underlined). The inserted base is shown in red. Bases following the deletion are shown in blue. *nbs ^2^* is a point mutation at nucleotide 2,113 (shown in red) that introduces a stop codon at amino acid position 686 (underlined). (B–E) Pharate adult morphology of wild-type (B and D) and *nbs^1^/nbs ^2^* (C and E) animals. *nbs^−^* pharate adults have a rough eye and missing bristle phenotype ([C and E], arrows). (F) *Drosophila* DNA damage response genes and their mammalian homologs. (G–X) p53- and Mnk-dependent apoptosis in *nbs* mutant wing discs. Wing imaginal discs from wild-type and *nbs* mutant third-instar larvae were mock treated or X-irradiated (4,000 rads) and stained with acridine orange (G–L) or with an antibody against cleaved caspase 3 ([M–R], apical sections and [S–X], basal sections). Wild-type untreated discs have very low levels of apoptosis (G, M, and S). *nbs* mutant discs have high levels of spontaneous apoptosis (I, O, and U). Irradiation of wild-type wing discs induces high levels of apoptosis (H, N, and T). Irradiation of *nbs* mutant discs (J, P, and V) does not greatly increase apoptosis beyond the elevated levels of spontaneous apoptosis (compare apical sections [N and P]). Apoptosis in *nbs* mutant discs is strongly suppressed by mutations in *p53* (K, Q, and W) and *mnk* (L, R, and X). The mutant alleles used in this figure and others are described in Materials and Methods.

One signature of telomeres that might contribute to HP1 and HOAP recruitment is the chromosome end itself. Studies in yeast and mammalian cells have demonstrated that telomere protection requires proteins that act at broken chromosome ends during the cellular response to DNA damage; these include the ATM and ATR protein kinases and the Mre11/Rad50/NBS1 (MRN) DNA repair complex [17,18]. Analysis of cells lacking telomerase and ATM suggests that ATM plays a particularly critical role in cells with short telomeres [19–22]. Such cells may be least able to utilize sequence-specific mechanisms for telomere protection. In both budding and fission yeast, the combined loss of the ATM and ATR pathways results in severe telomere protection defects [20,22–24]. In mammalian cell culture, acute inhibition of the MRN complex or of the ATM and ATR kinases also induces telomere fusions [25]. *Drosophila* homologs of most DNA damage response genes have been described (see Figure 1 for summary). The *Drosophila telomere fusion (tefu)* gene is required to prevent fusions in proliferating cells and is encoded by the *Drosophila* homolog of *ATM* [26,27]. Mutations in the *Drosophila* DNA damage response genes *tefu, mre11,* and *rad50* lead to partial loss of telomere protection and reduced recruitment of HP1 and HOAP to telomeres [26–31]. Thus, a DNA damage response pathway contributes to the protection of *Drosophila* telomeres; however, HP1 and HOAP can also mediate some degree of telomere protection in the absence of this pathway (see discussion in Oikemus et al. [27]).

Here, we characterize the role of Nbs and the ATM and ATR DNA damage response pathways in the epigenetic protection of *Drosophila* telomeres. In humans, mutations in Nbs1 or ATM result in similar inherited syndromes [32]. In both mammals and yeast, Nbs1 forms a complex with Mre11 and Rad50 (the MRN complex) that acts in the ATM pathway in response to DNA damage and is required for DNA repair and telomere function [33,34]. We demonstrate that Drosophila nbs is required for *atm-* and *atr*-dependent DNA damage responses including DNA repair. *Drosophila mei-41* (the *ATR* homolog) and *mus304* (the *ATRIP* homolog) act in parallel to the *atm* pathway in telomere protection; cells lacking both pathways fail to recruit HOAP to the telomeres of mitotic chromosomes and exhibit a severe telomere fusion phenotype. The telomere fusion defect in *nbs* mutants suggests that it acts in both the *tefu* and *mei-41-mus304* telomere protection pathways and in the chromosome joining step. We have taken advantage of the severe telomere fusion phenotype in cells lacking both pathways to test the role of DNA damage response pathways in the sequence-independent protection of *Drosophila* telomeres. Analysis of these cells reveals that loss of telomeric HOAP and telomere fusions are not due to loss of telomeric sequences. Furthermore, these DNA damage response pathways are also required to protect the telomeres of terminally deleted chromosomes, directly demonstrating that the DNA damage response pathways are required for epigenetic regulation of telomere protection.

## Results/Discussion

### *Drosophila* Nbs Is Required for Normal Development

To identify genes that cooperate with *atm/tefu* in telomere protection, we characterized mutations in other *Drosophila* DNA damage response genes including *nbs*. Figure 1F lists several *Drosophila* DNA damage response genes and their mammalian homologs. Similar to *nbs* homologs in other organisms, Drosophila nbs encodes a protein with N-terminal FHA and BRCT domains and a short region of similarity to the Mre11 interaction domain encoded by human *Nbs1* (Figure 1A). To identify mutations in *Drosophila nbs,* we screened a collection of lethal mutations in the genetic region containing *nbs* [35] for pupal lethality and excess apoptosis during wing development, phenotypes previously described for *Drosophila tefu, mre11,* and *rad50* [26,27,29–31]. Two mutations with these phenotypes failed to complement each other and their lethality was rescued by a transgene containing the *nbs* genomic region (Figure 1A; Materials and Methods). Sequencing of these mutations revealed that *l(3)67BDp^1^ (nbs^1^)* contains a 238–base pair (bp) deletion and 1bp insertion that disrupts the open reading frame while *l(3)67BDr^1^ (nbs^2^ )* introduces a stop codon that truncates the reading frame at amino acid position 685 (Figure 1A). Both of these mutations are predicted to eliminate the ability of Nbs to interact with Mre11.

Flies homozygous for the *nbs^1^* mutation die as pharate adults with rough eyes and missing or abnormal bristles (Figure 1B–1E). In *tefu, mre11,* or *rad50* mutant flies, this phenotype is accompanied by increased genomic instability and apoptosis [26,27,29–31]. *tefu,* but not *mei-41* or *mus304,* is also required for rapid induction (within 4 h) of additional apoptosis by X-irradiation [27,36]. The developing wings of *nbs* mutant animals also exhibit high levels of spontaneous apoptosis compared to wild-type animals (Figure 1G, 1I, 1M, 1O, 1S, and 1U). X-irradiation of these discs does not induce the rapid, large increase in apoptosis observed in wild-type discs (Figure 1J, 1P, and 1V). These results suggest that *nbs* acts in the *tefu* DNA damage response pathway to regulate apoptosis. Consistent with this conclusion, *nbs tefu* double mutant animals also exhibit high levels of apoptosis and fail to induce further apoptosis following irradiation (Figure S1).

To determine whether the elevated spontaneous apoptosis in these discs requires *p53* or *mnk* (the *Drosophila Chk2* homolog), apoptosis was examined in *nbs p53* and *nbs mnk* double mutant discs. The *Drosophila p53* and *mnk* genes are required for induction of apoptosis by X-irradiation [36–40]. Previously, the *Drosophila p53* gene was shown to be required for some, but not all, of the apoptosis observed in *tefu* mutant discs [27,30]. Apoptosis is substantially reduced in *nbs p53* (Figure 1K, 1Q, and 1W) and *nbs mnk* (Figure 1L, 1R, and 1X) double mutant discs compared to *nbs* single mutants (Figure 1I, 1O, and 1U). Although *p53* has been implicated in a variety of stress response pathways, *Chk2* homologs appear to specifically function in DNA damage responses. Thus, these results suggest that the absence of *nbs* leads to apoptosis via activation of a DNA damage response. This response may be directly activated by unprotected telomeres or by chromosome breaks formed following telomere fusions. The regulation of this response must, however, differ from the regulation of apoptosis 4 h following X-irradiation, which requires wild-type *nbs* and *tefu* function.

### DNA Damage Checkpoint and Repair Defects in *nbs* Mutant Cells

**Figure 2:**
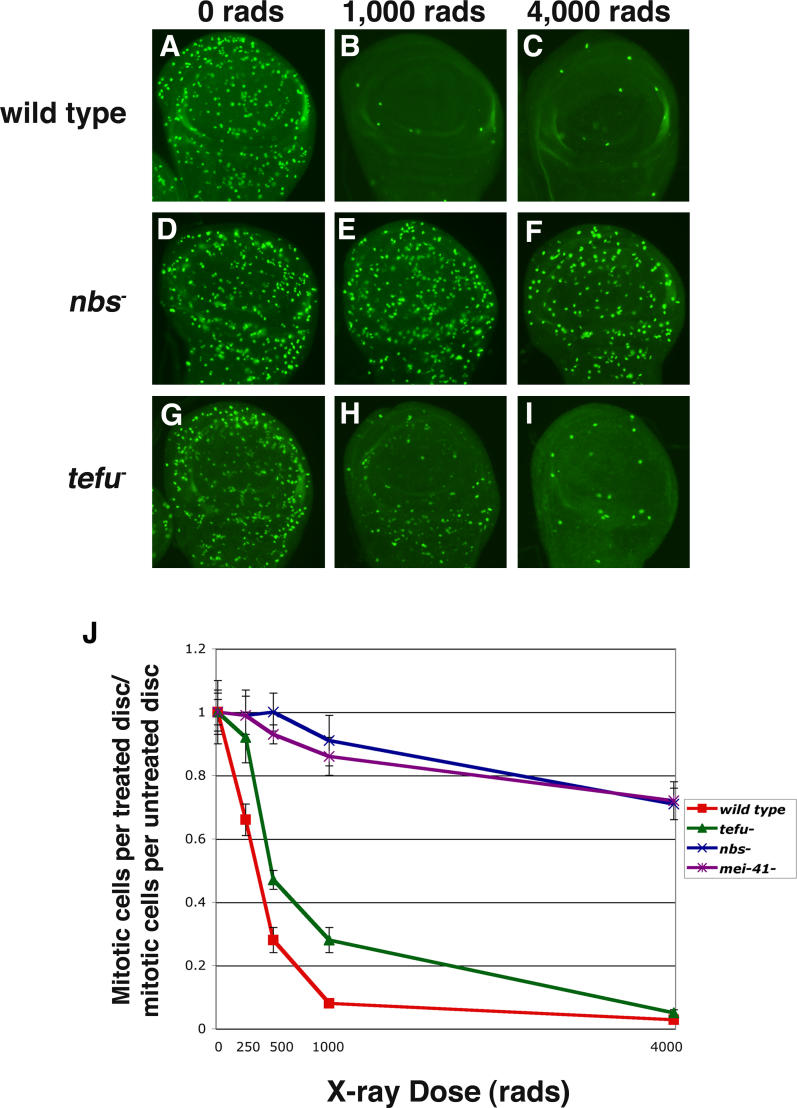
*Drosophila* Nbs Is Required for Damage-Induced Cell Cycle Arrest Third-instar larval wing discs were mock treated or treated with various doses (250, 500, 1,000, and 4,000 rads) of X-rays and then stained with an antibody against phosphorylated histone H3. (A–I) The pattern of mitotic cells in untreated and irradiated wild-type (A–C), *nbs* mutant (D–F), and *tefu* mutant (G–I) larval wing discs are shown. At 1,000 and 4,000 rads, mitosis is blocked in wild-type wing discs (B and C) whereas *nbs* mutant discs fail to arrest (E and F). *tefu* mutant wing discs have a partial mitotic arrest following treatment with 1,000 rads (H). At 4,000 rads, mitosis is completely blocked in *tefu* mutant wing discs (I). (J) The ratio of mitotic cells in wild-type, *nbs, tefu,* and *mei-41* mutant wing discs following X-irradiation to the number of mitotic cells in untreated discs of the same genotype is shown. Error bars indicate the standard error of the mean.

To further compare the function of *nbs* with *tefu, mei-41,* and *mus304,* cell cycle arrest and double-strand DNA break repair were examined. Previous studies have demonstrated that *mei-41* is required for G2 arrest at both high (4,000 rads) and low (500 rads) doses of ionizing radiation [41,42], whereas *tefu* is primarily required at low doses [26,43], but not high doses [27,30,31]. Dose-response curves confirm that *mei-41* mutant discs fail to arrest in response to a range of irradiation doses whereas *tefu* mutant discs have a partial arrest phenotype at low doses, but not at 4,000 rads (Figure 2). Similar to *mei-41, nbs* is required for cell cycle arrest at all doses tested (Figure 2). These results demonstrate that *nbs* plays a *tefu*-independent role in cell cycle arrest and suggests that it acts in the *atr-atrip* pathway to mediate G2 arrest. A cell cycle arrest defect at low, but not high X-ray doses has been reported for *tefu* and *mre11* mutant cells [43]; however, we observe that loss of *nbs* results in an arrest defect at high doses whereas loss of *mre11* results in a partial arrest at high doses (Table S1). *nbs mus304* and *nbs tefu* double mutants also exhibit a cell cycle checkpoint phenotype at high doses; however, the reduced number of mitotic cells and smaller discs indicates that mitosis has been severely disrupted in the double mutants, making direct comparisons to single mutants problematic (Table S1, Figure S2). We conclude that *nbs, mei-41,* and *mus304* are all essential for cell cycle arrest at high doses of X-irradiation whereas *tefu* is not.

**Figure 3:**
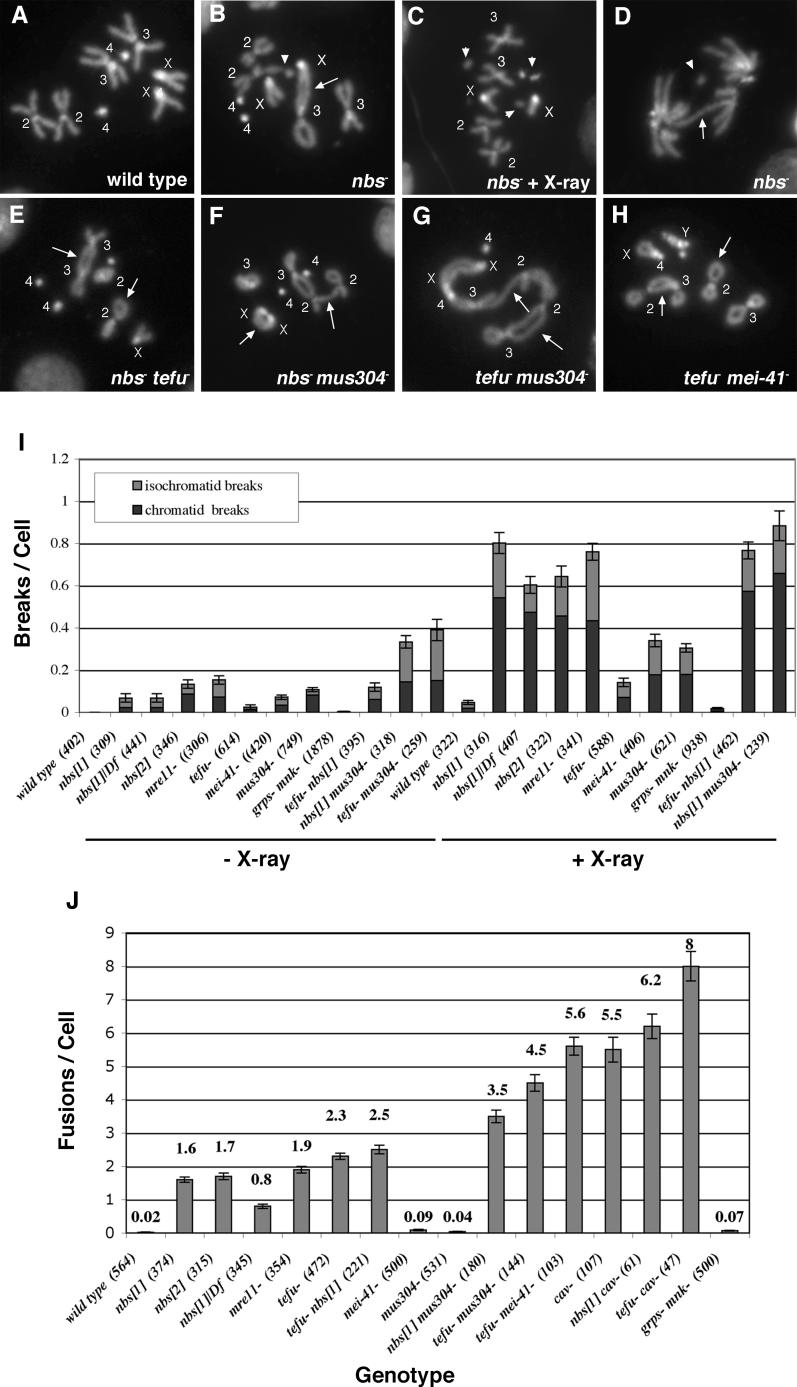
Drosophila nbs Acts with the *tefu* and *mei-41-mus304* Pathways to Protect Cells from Telomere Fusions and Chromosome Breaks Mitotic chromosome spreads were prepared from wild-type and mutant third-instar larval brains. Wild-type cells do not exhibit telomeric associations (A). *nbs* single mutant cells and *nbs tefu* double mutant cells exhibit DNA breaks (arrowheads) and telomere fusions (arrows) in metaphase (B, C, and E) and anaphase (D). Double mutant cells disrupting both the *atm-nbs* and *atr-atrip* pathways result in a more severe telomere phenotype, in which many telomeres are fused (F–H). The labels X, Y, 2, 3, and 4 refer to the relevant chromosome. The frequency of spontaneous (no X-ray treatment [− X-ray]) and damage-induced (treated with 100 rads X-rays [+ X-ray]) chromosome breaks is elevated in mitotic cells from *nbs* ^−^ and other mutant animals (I). Both isochromatid (light shading) and chromatid (dark colored portion of the bar) breaks were counted as one break. The frequency of telomere fusions per cell is elevated in DNA damage response mutant cells (J). Double telomere associations were counted as two fusions. Individual genotypes are discussed in the Results section. The total number of cells scored for each genotype is in parenthesis. Error bars indicate the standard error of the mean.

Previous studies have demonstrated that *mei-41, mus304, rad50,* and *mre11* are all required for DNA double-strand break (dsb) repair in *Drosophila* [28,29,42,44]. The effect of *nbs* and *tefu* mutations on dsb formation and repair was examined in metaphase chromosomes from larval neuroblasts (Figure 3). As discussed in more detail below, dsbs can arise as an indirect result of telomere fusion followed by chromosome breakage during mitosis. Broken chromosomes generated by this mechanism will generally retain their centromere. To analyze breaks due to mechanisms other than telomere fusion, the number of acentric chromosome fragments was analyzed. In untreated cells, these fragments may reflect a role in preventing formation of breaks during DNA replication. Both *nbs* and *tefu* are required to prevent the spontaneous accumulation of dsbs during normal cell cycles (Figure 3B, 3D, and 3I). However, *nbs, mre11, mei-41,* and *mus304* mutant cells all have a more severe phenotype than *tefu* (Figure 3I). Analysis of double mutant cells suggests that *nbs* and *tefu* act in parallel to *mus304* to prevent accumulation of dsbs (Figure 3I). *nbs* mutant cells also exhibit defective repair of X-irradiation–induced chromosome breaks (Figure 3C and 3I), consistent with the role of Nbs in the MRN DNA repair complex and with previous analysis of *Drosophila* Mre11 and Rad50 [28,29,44]. Less severe dsb repair defects are seen following X-irradiation of *tefu, mei-41,* or *mus304* mutant cells (Figure 3I). Following irradiation, *nbs tefu* or *nbs mus304* double mutant cells do not exhibit a greater defect than *nbs* single mutants, suggesting that *nbs* acts in both the *tefu* and *mus304* pathways to mediate repair of induced DNA breaks.

In summary, *nbs* acts in both the *tefu* and *mei-41-mus304* DNA damage response pathways. Double mutant analysis indicates that Drosophila nbs acts in common genetic pathways with *tefu* and *mus304* during DNA repair (Figure 3). In addition, *nbs* has DNA damage response phenotypes in common with both *tefu* (defective induction of apoptosis, Figure 1) and *mei-41-mus304* (defective induction of cell cycle arrest at high doses of X-irradiation, Figure 2). Although Nbs1 homologs are best known for their roles in DNA repair and signaling in the ATM pathway [32], human Nbs1 is also required for signaling by the ATR pathway [45]. Thus, *nbs* has a conserved role in *ATM-* and *ATR-ATRIP*–dependent DNA damage responses.

### Two DNA Damage Response Pathways Contribute to Telomere Protection

Metaphase larval neuroblasts were also used to examine the roles of different DNA damage response genes in telomere protection. Previous studies have demonstrated that *Drosophila tefu, mre11,* and *rad50* mutant cells have a partial defect in telomere protection [26–31]. Consistent with these results, *nbs* mutant animals exhibit a high frequency of cells with one or more fusions (Figure 3). These fusions are observed during both metaphase (Figure 3B and 3J) and anaphase (Figure 3D and Table S2). Another group has also recently described a telomere fusion phenotype for *nbs^1^* animals [46]. *nbs tefu* double mutant cells exhibit similar fusion rates as *tefu* single mutants, indicating that these genes act in a common telomere protection pathway (Figure 3E and 3J). These results are consistent with results in *Drosophila* and other organisms, indicating that ATM and components of the MRN complex act in a common telomere protection pathway [17,29]. Downstream targets of ATM in the mammalian DNA damage response pathway include Nbs1 and the checkpoint kinases CHK1 and CHK2. The *Drosophila* homologs of these kinases are encoded by the *grp* and *mnk* genes and are required for DNA damage-induced apoptosis and cell cycle arrest [36,47] . Both telomere protection and chromosome break repair are normal in *grp mnk* double mutant cells (Figure 3I and 3J), indicating that other targets of Drosophila tefu and *nbs* are responsible for their telomere protection and DNA repair functions.

Compared with mutations in the genes that encode the telomere protection proteins HOAP (Figure 3J, *cav^−^*) and HP1, mutations in *tefu, nbs, mre11,* and *rad50* exhibit a significantly lower frequency of telomere fusions [12,13,26–31], indicating that there may be a *tefu-nbs*–independent pathway for telomere protection. In mammals, the ATR checkpoint kinase is recruited to sites of DNA damage by ATRIP [48,49] and acts in parallel to the ATM kinase in the DNA damage response [32]. In budding and fission yeast, disruption of both *atm/atr* homologs results in loss of telomere protection [23,24]. Mutations in *mei-41* or *mus304* do not result in telomere protection defects (Figure 3J). However, *nbs mus304, tefu mus304,* and *tefu mei-41* double mutant animals all show higher rates of telomere fusion than the corresponding single mutants (Figure 3F, 3G, 3H, and 3J) indicating that the *mei-41-mus304* pathway acts in parallel to a *tefu-nbs* pathway to mediate telomere protection. The higher fusion frequency in *tefu mei-41* double mutants compared with *tefu mus304* double mutants may indicate that there is a small amount of *mei-41* activity in the absence of *mus304.*

### DNA Damage Response Genes Regulate Telomeric HOAP

The formation of telomere fusions requires two steps: (1) the failure of telomeric protein complexes, such as HP1-HOAP, to prevent telomeric DNA ends from being recognized as damage-induced ends and (2) the subsequent ligation of unprotected telomeres by DNA repair systems. To probe the role of DNA damage response pathways in the first step, HOAP localization was examined in individual mitotic cells (neuroblasts). Previously, it was shown that levels of the telomere protection proteins HP1 and HOAP are reduced at the telomeres of polytene chromosomes from *tefu* salivary gland cells [27], but that telomeric HOAP is not strongly reduced in mitotic chromosomes from neuroblasts [27,29]; these results suggest that in the absence of *tefu,* neuroblasts utilize an alternative mechanism for HOAP localization. In contrast, both salivary glands and neuroblasts required *mre11* and *rad50* for normal HOAP localization [28,29].

**Figure 4:**
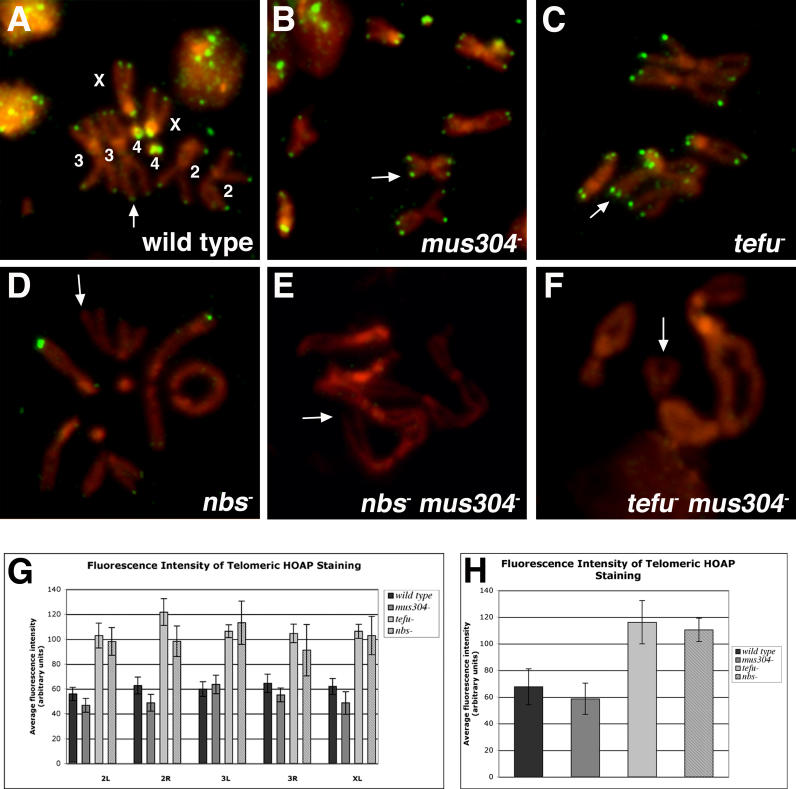
The Drosophila tefu and *mei-41-mus304* Pathways Are Required for Telomeric Localization of HOAP HOAP immunostaining of mitotic chromosomes prepared from wild-type and mutant third-instar larval brains. Wild-type, *mus304,* and *tefu* mutant mitotic chromosomes exhibit HOAP localization (shown in green) at most telomeres ([A–C] arrows). *nbs* mutant cells exhibit a decreased number of telomeres with HOAP signal ([D] arrow). No HOAP was detected at the telomeres of *nbs mus304* or *tefu mus304* mutant chromosomes ([E and F] arrows). Alleles examined in these experiments include (C) *tefu^1^/ Df(3R)PG4* and (E) *tefu^Δ356^ mus304^D2^*. The frequency of HOAP-positive telomeres is shown in Table 1. The labels X, 2, 3, and 4 refer to the relevant chromosome. (G) The average fluorescence intensity of anti-HOAP immunostaining at the telomeres of chromosome arms 2L, 2R, 3R, 3L, and XL was determined for wild-type, homozygous *mus304* ^−^ *, tefu^1^/Df(3R)PG4,* or *nbs* ^−^ animals. The average fluorescence intensity of the HOAP signal is higher in *tefu* and *nbs* mutant cells compared to wild-type or *mus304* mutant cells. (H) The average fluorescence intensity of HOAP staining at all telomeres from wild-type and mutant cells. Error bars indicate the standard error of the mean.

The frequency of neuroblast telomeres with HOAP staining and the intensity of staining at those telomeres were examined in wild-type and mutant cells (Figure 4, Table 1). Measurements of fluorescence intensity can be used to demonstrate that HOAP levels at individual telomeres are reproducibly increased or decreased in different genotypes. (However, we note that there may not be a linear relationship between the percent change of fluorescence observed and the percent change of telomeric HOAP protein levels.) Most wild-type, *tefu,* or *mus304* mutant metaphase cells are HOAP positive; between 77% and 94% of these cells had HOAP signals at chromosome ends (Figure 4, Table 1). Among the HOAP positive cells, between 66% and 72% of telomeres stained for HOAP. The fluorescence intensity of HOAP staining was similar at the telomeres of each of the major chromosome arms in both wild-type and *mus304* mutant cells (Figure 4G and 4H). However, the average intensity of HOAP staining was elevated in *tefu* mutant cells (Figure 4G and 4H), indicating that although HOAP is still recruited to telomeres, the mechanism regulating HOAP levels at telomeres may be perturbed. A more severe effect on HOAP localization was observed in *nbs* mutant metaphases, with only 44% of metaphases displaying HOAP signals and only 30% of the telomeres in those cells staining for HOAP (Table 1). This phenotype is similar to that reported for *mre11* and *rad50* mutant neuroblasts [28,29]. At the few HOAP-positive telomeres that are present in *nbs* mutant cells, HOAP fluorescence staining intensity was elevated compared to wild type, similar to the HOAP staining at *tefu* mutant telomeres (Figure 4H). Together with the genetic data indicating that *tefu* and *nbs* act in a common telomere protection pathway, these results suggest that an alternative pathway can maintain HOAP levels at telomeres, but that this pathway is much less efficient in *nbs* mutant cells.

**Table 1:**
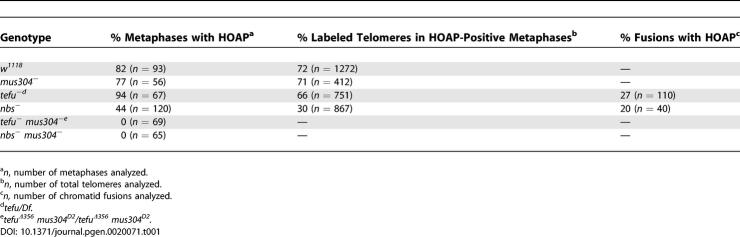
Quantification of HOAP Telomeric Signals

Since *mus304 nbs, mus304 tefu,* and *mei-41 tefu* double mutant cells have more severe telomere fusion phenotypes than *nbs* or *tefu* single mutants (Figure 3), the *mei-41-mus304* pathway is a clear candidate to recruit HOAP to telomeres in the absence of *tefu* or *nbs*. *mus304* single mutant animals do not exhibit a defect in either the frequency of HOAP-positive telomeres or the intensity of HOAP staining at those telomeres (Figure 4B and 4G and Table 1). In contrast, we were unable to detect telomeric HOAP staining in *mus304 tefu* or *mus304 nbs* double mutant cells (Figure 4E and 4F and Table 1). Thus, the *mei-41-mus304* pathway partially compensates for the absence of *tefu,* limiting the severity of the *tefu* telomere fusion phenotype. Cells lacking both pathways exhibit loss of telomeric HOAP and a severe telomere fusion phenotype. In a report published while this work was in preparation, Bi et al. also find that disruption of the Drosophila atm and *atr* pathways results in a high frequency of telomere fusions and loss of telomeric HOAP [46].

These results support the model that the Drosophila tefu and *mei-41-mus304* DNA damage response pathways mediate telomere protection by recruiting or maintaining HOAP at telomeres. The more severe HOAP localization phenotype of *nbs* mutant cells compared with *tefu* mutant cells indicates that *nbs* has a *tefu-*independent role in telomere protection. As described above, the common DNA repair and damage response phenotypes of *nbs* with *mei-41* and *mus304* indicate that *nbs* also acts in the *mei-41-mus304* DNA damage response pathway. Thus, one explanation for the lower frequency of HOAP positive telomeres in *nbs* compared to *tefu* mutant cells is that *nbs* mutations both disrupt the *tefu* telomere protection function and partially disable a compensatory telomere protection pathway mediated by *mei-41-mus304*.

There is a good correlation between the levels of telomeric HOAP and the frequency of telomere fusion, except in *nbs, mre11,* and *rad50* mutants. In yeast and mammalian cells, some DNA repair genes are required to both maintain telomere protection and to promote joining of unprotected telomeres [17,50]. The observed telomere fusion frequency in *nbs* mutant cells may reflect the combined effects of decreased telomere protection and inefficient fusion of unprotected telomeres. Although the loss of *nbs* has a more severe effect than *tefu* on telomeric HOAP (Figure 4), *nbs* and *tefu* mutant cells have similar telomere fusion frequencies (Figure 3). *nbs* mutations have a more severe effect on repair of DNA breaks (Figure 3), suggesting that *nbs* mutant cells may also have reduced joining of unprotected telomeres. Consistent with a role for *nbs* in fusion of unprotected telomeres, *nbs mus304* mutant cells have a lower telomere fusion frequency than *tefu mus304* mutant cells, despite undetectable levels of telomeric HOAP in both genotypes. Similarly, *nbs cav* double mutant cells have a lower telomere fusion frequency than *tefu cav* double mutant cells (Figure 3J).

In summary, DNA damage response genes are essential for the telomeric localization of the protection protein HOAP. Analysis of DNA repair, telomere fusions and HOAP localization suggests that the telomere fusion frequency reflects a combination of defective protection and reduced fusion of unprotected chromosomes. Although these results do not rule out the possibility that DNA damage response genes are also required for modification of HP1 and HOAP complexes at telomeres, they strongly suggest that recruitment or maintenance of these complexes to telomeres is critical for telomere protection.

### DNA Damage Response Pathways Are Required for Epigenetic Protection of Telomeres

In many organisms, telomere-specific sequences are required to recruit proteins that prevent chromosome end fusion. Loss of these sequences in cells that do not express telomerase or that are mutant for DNA damage response genes can result in telomere fusions. However, in *Drosophila,* the stable protection of terminally deleted chromosomes from telomere fusion suggests that a sequence-independent mechanism acts to protect the telomeres of normal chromosomes [4–8]. Given the requirement of the *tefu* and *mei-41-mus304* DNA damage response pathways for telomere protection, we propose that recognition of chromosome ends contributes to this epigenetic phenomenon. One prediction of this model is that cells lacking these pathways will exhibit telomere fusion without loss of telomeric DNA sequences such as *HeT-A*. *HeT-A* sequences should not be lost simply as a secondary effect of unprotected telomeres since telomere fusions in cells lacking HP1 function still retain these sequences [13]. A second prediction is that terminal deletion chromosomes lacking telomeric sequences will still fuse in the absence of the DNA damage response pathways. This observation would rule out the possibility that the epigenetic mechanism for protection of terminal deletions utilizes an alternative mechanism to recruit HP1 and HOAP that is independent of the DNA damage response pathways. These predictions can be evaluated in animals with the extreme telomere fusion phenotype associated with loss of both the *tefu* and *mei-41-mus304* DNA damage response pathways.

**Figure 5:**
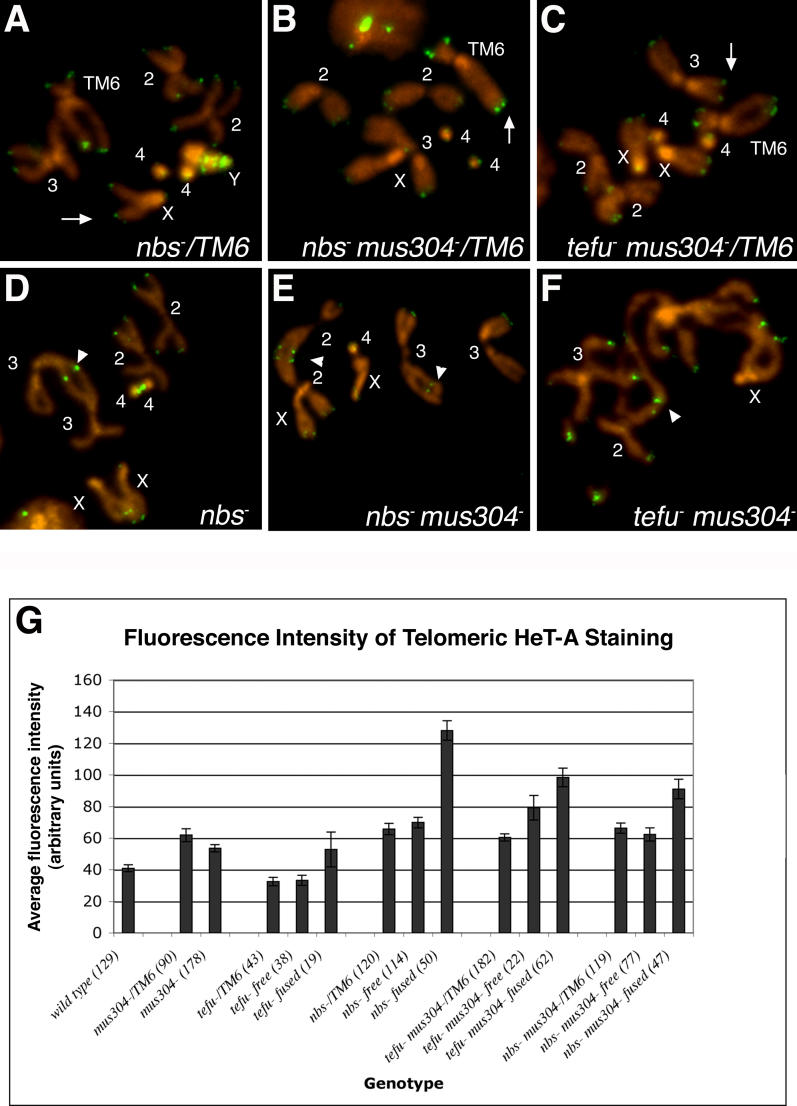
Loss of Telomeric Sequences Is Not Required for Telomere Fusions in Cells Lacking the *tefu* and *mei-41-mus304* DNA Damage Response Pathways (A–F) Fluorescent in situ hybridization of mitotic chromosomes from third-instar larval brains with a *HeT-A* probe. Telomeric *HeT-A* sequences are present at the free ends (arrows) and telomere fusion sites (arrowheads) of *nbs^1^* (D) *nbs^1^ mus304^D2^* / *nbs^1^ mus304^D1^* (E) and *tefu^1^ mus304^D2^* (F) homozygous mutant chromosomes. Similar levels of *HeT-A* hybridization are seen at the telomeres of the corresponding heterozygous chromosomes (A, B, and C). Internal sites of *HeT-A* adjacent to a chromosome end mark sites of telomere fusion followed by chromosome breakage ([E], Chromosome 3, arrowhead). Pairs of internal *HeT-A* sites mark sites where breakage was followed by a second fusion event ([E], Chromosome 2, arrowhead). The frequency of *HeT-A* positive telomeres is shown in Table 2. The labels TM6, X, Y, 2, 3, and 4 refer to the relevant chromosome. (G) The average fluorescence intensity of *HeT-A* signal at both free ends and fused telomeres of homozygous and heterozygous mutant animals was determined following fluorescent in situ hybridization. The fluorescence intensity of *HeT-A* signals is similar or greater in homozygous mutant cells compared with the corresponding heterozygous cell. For a given genotype, the fluorescence intensity of *HeT-A* signals is similar or greater at sites of chromosome fusions compared to free chromosome ends. Error bars indicate the standard error of the mean. The number of telomeres scored for each genotype is indicated in parenthesis.

To test the first prediction, the telomere-specific retrotransposon *HeT-A* was analyzed at individual telomeres of DNA damage response defective cells by fluorescence in situ hybridization. Measurements of fluorescence intensity can be used to demonstrate that *HeT-A* levels at individual telomeres are reproducibly increased or decreased in different genotypes. (However, we note that there may not be a linear relationship between the percent change of fluorescence observed and the percent change of telomeric *HeT-A* DNA.) Previously, telomere fusions in *tefu* mutant cells were shown to retain at least some *HeT-A* sequences [27,29]. However, these studies only examined mutants with mild telomere fusion phenotypes and were less thorough than the analysis presented here. Because the number of *HeT-A* copies per telomere can vary between strains, particularly in strains with altered HP1 function [51], *HeT-A* signals at free chromosome ends in homozygous mutant animals were compared to chromosome fusion sites in the same cells and to free chromosome ends in an appropriate heterozygous parental strain (Figure 5, Table 2). *HeT-A* is still present at free telomeres and at chromosome fusion sites in *tefu, nbs, tefu mus304,* and *nbs mus304* homozygous mutant cells (Figure 5A–5F and Table 2). For each genotype, both the frequency and intensity of *HeT-A* staining at chromosome fusions is equal to or greater than that observed at the free chromosome ends (Table 2 and Figure 5G), indicating that loss of telomere-specific sequences does not correlate with telomere fusion in cells with defective DNA damage response pathways. Note that if a *HeT-A*–positive telomere fuses with another *HeT-A–*positive telomere, the intensity of staining will increase; if it fuses with a *HeT-A*–negative telomere or a chromosome break, the intensity should be the same. Different genotypes exhibit different relative intensities of *HeT-A* staining at chromosome fusions compared to free ends (Figure 5G). These differences may reflect different frequencies of telomere–telomere fusions versus telomere–break fusions or differences in the precise mechanism of telomere fusion. Nonetheless, the observation that the staining intensity at fusions is equal to or greater than the intensity at free chromosome ends demonstrates that loss of these sequences is not required for fusion in any of these genotypes.

**Table 2:**
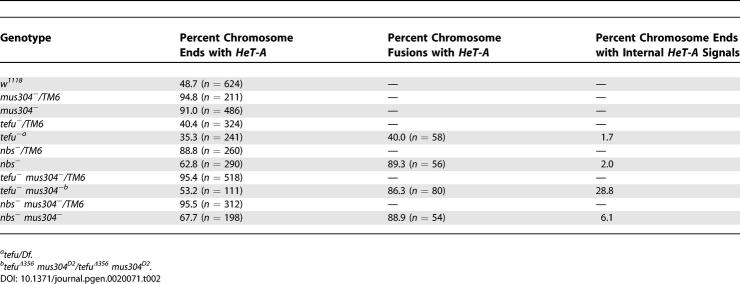
Percent Chromosomes with *HeT-A* Staining

The frequency and intensity of *HeT-A* staining was also compared at the free chromosome ends of mutant cells and the corresponding parental strain. The frequency of *HeT-A* staining at chromosome ends in homozygous *nbs, nbs mus304,* and *tefu mus304* mutant cells is lower than in cells from the corresponding heterozygous strains (Table 2). Although this decrease could reflect removal of telomeric sequences in homozygous mutant animals, two other factors are likely to contribute. First, defective DNA repair generates chromosome ends without telomeric sequences. As demonstrated above (Figure 3), several of these mutations result in high levels of spontaneous breaks. Second, progression of cells with telomere fusions through mitosis generates anaphase bridges and chromosome breaks via the fusion/bridge/break cycle. In one example (Figure 5E, arrowhead), an internal site of *HeT-A* (the original fusion site) is adjacent to a chromosome end without *HeT-A* (the break site). Chromosome ends with adjacent internal *HeT-A* sites are found in all mutant cells with telomere fusions (Table 2). The overall frequency of breaks resulting from fusion is underestimated by this analysis since some broken chromosomes will not include the original fusion site. Thus, chromosome breaks can account for the increased number of ends without *HeT-A* staining. However, at those chromosome ends that are *HeT-A* positive, the intensity of staining is equal to or greater than in the corresponding heterozygous cells (Figure 5G). Combined with the analysis of *HeT-A* staining at fusion sites described above, these results indicate that the fusion phenotype of single or double mutants in the DNA damage response pathways is not due to loss of telomeric sequences.

**Figure 6:**
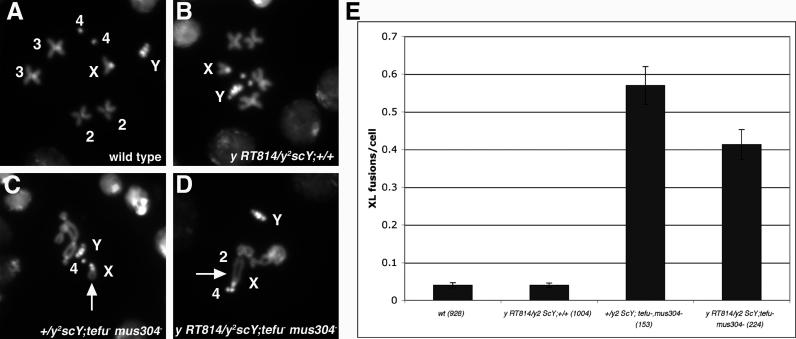
The Drosophila tefu and *mei-41-mus304* Pathways Act in an Epigenetic Telomere Protection Mechanism Normal (A) and terminally deleted (B) X chromosomes are not fused in wild-type cells. Normal (C) and terminally deleted X chromosomes (D) are fused in *tefu mus304* double mutant cells. Both sister chromosome fusions (C) and non-sister fusions (D) are observed. High frequencies of X chromosome telomere fusions per cell are observed for normal and terminally deleted chromosomes in *tefu mus304* mutant cells (E). Error bars indicate the standard error of the mean. The number of cells scored for each genotype is in parenthesis. The labels X, Y, 2, 3, and 4 refer to the relevant chromosome.

A second prediction of the end-recognition model for *Drosophila* telomere protection is that both normal and terminally deleted chromosomes will exhibit similar frequencies of fusion in cells lacking the DNA damage response pathways. The stable protection of terminally deleted chromosomes in wild-type cells suggests that the telomeres of normal chromosomes are also protected by sequence-independent mechanism; however, it is also possible that terminally deleted chromosomes acquire an alternative mechanism for telomere protection and that the DNA damage response pathways must act in conjunction with a sequence-specific mechanism. To address this possibility, we examined fusion rates of a normal and a terminally deleted X chromosome in *tefu mus304* double mutant cells. Previous experiments have demonstrated that the telomere protection gene *UbcD1* is required to prevent fusion of terminally deleted chromosomes [52]. In *tefu mus304* double mutant cells, a normal and a terminally deleted X chromosome fused to the sister or to heterologous chromosomes at a high frequency (Figure 6). The fusion frequency is similar but lower than observed with a normal X chromosome (*p* = 0.019, two-tailed Fisher Exact Test); this difference may indicate that the terminally deleted chromosome is slightly less sensitive to the loss of DNA damage signaling pathways. Nonetheless, the frequent fusion of terminally deleted chromosomes in *tefu mus304* double mutant cells directly demonstrates that the DNA damage response pathways act in an epigenetic mechanism for telomere protection.

### Concluding Remarks

DNA damage response genes have evolutionarily conserved roles in telomere function. Unprotected telomeres are recognized by these pathways and elicit a variety of cellular responses including apoptosis and end-to-end fusion of chromosomes. However, these same pathways are also required to promote telomere protection. We demonstrate that the *Drosophila atm* and *atr-atrip* DNA damage response pathways act in an epigenetic mechanism to mediate telomere protection. In cells lacking both pathways, the chromatin-associated protein HOAP is not recruited to telomeres and both normal and terminally deleted chromosomes undergo fusion at a high frequency. Furthermore, fusion of normal telomeres occurs without loss of telomere-specific sequences. Taken together, these results support an end-recognition model in which DNA damage response proteins recognize a DNA structure at the chromosome end and recruit or stabilize the telomere protection proteins HP1 and HOAP at telomeres; in turn, these proteins act to prevent the ligation of chromosome ends by DNA repair enzymes and the activation of p53-dependent apoptosis. In other organisms, a similar epigenetic mechanism may act in conjunction with sequence-specific protection mechanisms or may be utilized to promote protection of critically short telomeres, which are least able to utilize sequence-specific binding proteins.

## Materials and Methods

### *Drosophila* strains and crosses.

All animals were raised at 25 °C. Mutations in *nbs* were identified from a collection of lethal mutations in the cytological region 67A-D [35]. To identify sequence alterations, genomic DNA from animals homozygous for *l(3)67BDp^1^* and *l(3)67BDr^1^* was amplified by PCR and sequenced. An *nbs* rescue construct was created by amplifying a genomic fragment containing the *nbs* transcript and 377 bp of upstream sequence and 102 bp of downstream sequence using the following primers: 5′GGCCAGATCTGGTCAGGTGAGACATGGGTTAC3′ and 5′GGCCGGTACCAGGAAACTGAATCCTCCTCC3′. The genomic fragment was cloned into the BglI and KpnI sites of the pUAST vector [53]. Flies carrying the P[UAS-*nbs*] rescue transgene were created by P-element–mediated germline transformation. Transgene rescue was tested by crossing *+/+; nbs^1^/TM6BTb* females to P[*nbs^+^*]/*CyO; Df(3L)Ac1/TM6BTb* males and scoring non-balancer animals.

The *w^1118^* strain was used as the wild-type stock. Where alleles are not otherwise indicated, the following alleles or allelic combinations were used: *mei-41^D3^; mnk ^p6^; grp ^fs1^; mre11^Δ^; tefu^Δ356^/tefu^1^; nbs^1^;* and *mus304^D1^/mus304^D2^*. Alleles are described in the text or at http://www.flybase.org. Double mutants were created by standard genetic crosses and confirmed by complementation analysis or PCR.

The terminally deleted X chromosome *y RT814* was originally generated by Dr. Jim Mason and was obtained from Dr. Maurizio Gatti. The deleted region includes the *yellow (y)* gene and all genes distal to it. In situ hybridization of polytene chromosomes with *HeT-A* and *TART* probes was performed to confirm that the *y RT814* chromosome did not terminate with a retrotransposon. To examine this chromosome in *tefu mus304* double mutant cells, *y RT814/+; tefu^Δ356/+^ mus304^D2/+^* females were crossed to *+/y^2^ scY; tefu^1/+^ mus304^D1/+^* males. Individual larval males carrying the terminally deleted X chromosome were identified by PCR analysis of the *yellow* gene using the following primers, which flank the *gypsy* transposon insertion in the *y^2^* allele: 5′ ATTGTGAATCATCGGTGACG 3′ and 5′ CATGCAGACAAAAATCCAGAAA 3′. Males with the deletion chromosome do not produce a PCR product because the X chromosome deletion removes the *y* gene and the Y chromosome carries the *y^2^* allele (Figure S3A). A second pair of primers to a different region of the *y* gene were used as a positive control to amplify a product in animals with either a normal or terminally deleted X chromosome (Figure S3B): 5′ CATGCAGACAAAAATCCAGAAA 3′ and 5′ ATTGTGAATCATCGGTGACG 3′. *tefu^−^ mus304^−^* homozygous animals were identified by their small imaginal disc size and confirmed by their chromosome fusion phenotype. In all cases, the disc size and chromosome fusion phenotype matched. The high frequency of fusions and small disc size was confirmed to be specific for brains homozygous for *tefu^−^ mus304^−^* and not for brains homozygous for *tefu^−^* and heterozygous for *mus304* (unpublished data).

### Apoptosis and checkpoint assay.

Late third-instar larvae were treated with 4,000 rads in a Faxitron RX650 (Faxitron X-ray Corporation, Wheeling, Illinois, United States). Apoptotic cells were detected in wing imaginal discs 4 h after irradiation as described previously [27]. Mitotic cells were visualized using a phospho-histone H3 antibody (Upstate Biotechnology). Fixation and staining were performed as previously described [42]. The number of mitotic cells per wing disc was determined by flattening mounted wing imaginal discs and counting the number of phospho-H3–positive cells. The number of mitotic cells at each X-ray dose was normalized to the average number of mitotic cells in untreated discs. At least five discs were analyzed for each genotype and dose.

### Fusion and break analysis.

Late third-instar larvae were treated with 100 rads in a Faxitron RX650 using an aluminum shield to block lower energy wavelengths. Larval brains were dissected 2.5 h following irradiation, and chromosome spreads were prepared as described previously [26]. Spontaneous and irradiation-induced breaks were quantified by counting chromatid and isochromatid breaks with acentric fragments.

### Immunostaining of mitotic cells.

Mitotic chromosomes were stained with a rabbit polyclonal anti-HOAP antibody (gift of R. Kellum, University of Kentucky). The HOAP antibody (1:200 dilution in 10% FBS, 1× PBS, 0.1% Tween) was preabsorbed with fixed embryos overnight at 4 °C. Neuroblast squashes were prepared as described [26] with the following changes: After hypotonic treatment, larval brains were fixed sequentially in formaldehyde solution (2% formaldehyde, 2% triton, 1× PBS) for 1 min and then in acetic acid/formaldehyde solution (2% formaldehyde, 45% glacial acetic acid in water) for 6 min. Slides were washed in PBST and incubated for 1 h at room temperature in blocking solution (10% FBS, 1× PBS, 0.1% Tween). Slides were incubated with anti-HOAP antibody overnight at 4 °C, then rinsed twice in PBST for 15 min and incubated for 1 h at room temperature with secondary antibody (anti-rabbit-Alexa 488, [Vector Laboratories, Burlingame, California, United States], diluted 1/2,000 in blocking solution). Finally, slides were washed twice in PBST for 15 min and mounted in Vectashield containing DAPI (Vector Laboratories). Chromosome preparations were observed using an Axiovert 200 Carl Zeiss microscope (Oberkochen, Germany), and mitotic figures were collected with the Axiovision 4.4 Zeiss software. Quantification of the HOAP fluorescence intensity was performed using the Image J software package (http://rsb.info.nih.gov/ij).

### Fluorescent in situ hybridization of *Drosophila* neuroblasts.

In situ hybridization to mitotic chromosomes was performed as described [54]. *HeT-A* probe was labeled with Biotin-14-dUTP using the Bionick Translation System (GIBCO BRL, Rockville, Maryland, United States). Slides were mounted in Vectashield containing DAPI as a DNA counterstain (Vector Laboratories). Chromosomes were identified through their specific peri-centromeric banding pattern after DAPI staining. Chromosome preparations were observed using an Axiovert 200 Carl Zeiss Microscope, and mitotic figures were collected with the Axiovision 4.4 Zeiss software. Quantification of the *HeT-A* fluorescence intensity was performed using the ImageJ software package.

## Supporting Information

Figure S1 Drosophila nbs and *tefu* Act in the Same Damage-Induced Apoptosis Pathway Third-instar larval wing discs were stained with acridine orange or an antibody against activated caspase 3 in order to visualize apoptosis. Wild-type untreated discs have very low levels of apoptosis (A), (E), and (I). *tefu nbs* (B), (F), and (J) and *nbs mus304* (D), (H), and (L) mutant discs have high levels of spontaneous apoptosis as seen in *tefu* and *nbs* single mutants. In addition, *nbs mus304* discs are small and misshapen compared to wild-type discs. Irradiation of *tefu nbs* double mutant discs does not result in any further increase in apoptosis (C), (G), and (K), similar to *tefu* or *nbs* single mutant discs. The mutant alleles used in this figure and others are described in Materials and Methods.
(9.9 MB TIF)

Figure S2 Double Mutant Animals Exhibit a Cell Cycle Arrest Defect and a Reduced Number of Mitotic Cells Larval wing discs were mock treated or treated with 4,000 rads and then stained with an antibody against phosphorylated Histone H3. The pattern of mitotic cells was examined in wild-type (A) and (C), *tefu nbs* (B) and (D), and *nbs mus304* (C) and (F) mutant larval wing discs. At 4,000 rads, mitosis is blocked in wild-type wing discs (D). However, *tefu nbs* (E) and *nbs mus304* (F) mutant cells fail to arrest following irradiation. A direct comparison to the single mutants was not possible due to the reduced size of the double mutant discs and the corresponding reduced number of mitotic cells (Table S1).
(2.7 MB TIF)

Figure S3 Identification of Individuals with Terminal Deletions by PCR PCR was performed using primers specific for the wild-type *yellow (y^+^)* gene (A) or with control primers that produce a product with either *y^+^* or *y^2^,* an allele with a transposon insertion in the *yellow* gene (B). Genomic DNA was isolated from a *w^1118^* male (wild-type control, lane 1) and from a *y RT814 / y^2^ sc Y* male (lane 2) that carries a terminally deleted X chromosome that deletes *y* and a Y chromosome carrying *y^2^* (lane 2). There is no product produced in the absence of *y^+^* (lane 2). PCR analysis of genomic DNA isolated from individual *tefu mus304* mutant male larvae from the cross, *yRT814/+; tefu^Δ356/+^ mus304^D2/+^* females mated to *+/y^2^ sc Y; tefu^1/+^mus304^D1^* ^/+^ males, distinguished animals carrying the terminally deleted X chromosome (lanes 4, 5, and 6) from those carrying a wild-type X chromosome (lanes 3, 7, 8, and 9).
(1.1 MB TIF)

Table S1 Average Number of Phospho-H3–Positive Cells per Wing Imaginal Disc (22 KB DOC)

Table S2 Anaphase Bridges in Drosophila nbs Mutants (22 KB DOC)

### Accession Numbers

The National Center for Biotechnology Information (NCBI) (http://www.ncbi.nlm.nih.gov) Entrez Gene IDs for the genes and gene products discussed in this paper are ATM (472), ATR (545), ATRIP (11277), Chk1 (1111), Chk2 (11200), *grps* (34993), *mei-41* (32608), *mnk* (35288), *mus304* (40003), *nbs* (44259), NBS1 (4683), *p53* (2768677), and *tefu* (41839).

## Abbreviations

bp: base pair
dsb: double-strand break
MRN: Mre11/Rad50/NBS1
