# Supplementary material for: Epigenetic Telomere Protection by Drosophila DNA Damage Response Pathways
Source: PLoS Genet. 2006 May 19;2(5):e71. doi: 10.1371/journal.pgen.0020071 (PMC1463044; doi:10.1371/journal.pgen.0020071)
Supplement: Supplementary file 1 [file pgen.0020071.st001.doc]

Table S1. Average number of phospho-H3 positive per wing imaginal disc

| Genotype | Average mitotic cells/disc untreated | Average mitotic cells/disc treated (4,000 Rads) |
| --- | --- | --- |
| *w^1118^* | 348  (+/-19.9) | 10  (+/- 2.8) |
| *mei-41^-^* | 386  (+/-17.3) | 278  (+/- 22.6) |
| *tefu^- (d)^* | 347  (23) | 18  (+/- 2.1) |
| *nbs^-^* | 389  (+/- 40.6) | 276  (+/- 20.9) |
| *mre11^-^* | 426  (+/- 12.1) | 169  (+/- 15.2) |
| *tefu^-^nbs^-^* | 257  (+/- 14.5) | 288  (+/- 22.1) |
| *nbs^-^ mus304^-^* | 150  (+/- 8.3) | 139  (+/- 7.1) |

Five to ten discs were scored for each genotype and condition. The standard error of the mean is indicated in parenthesis.
