# Supplementary material for: Epigenetic Telomere Protection by Drosophila DNA Damage Response Pathways
Source: PLoS Genet. 2006 May 19;2(5):e71. doi: 10.1371/journal.pgen.0020071 (PMC1463044; doi:10.1371/journal.pgen.0020071)
Supplement: Supplementary file 2 [file pgen.0020071.st002.doc]

Table S2. Anaphase Bridges in Drosophila *nbs* Mutants

| Genotype | Nunber of anaphases | Number of anaphase bridges^(a)^ | Number of broken anaphases^(b)^ |
| --- | --- | --- | --- |
| *w^1118^* | 203 | 1 (0.5) | 0 |
| *w^1118^ / Df(3L)AC1* | 165 | 1 (0.6) | 1 (0.6) |
| *nbs^1^ / nbs^1^* | 140 | 75 (54) | 10 (7.1) |
| *nbs^1^ / Df(3L)AC1* | 226 | 120 (53) | 62 (27) |
| *nbs^2^ / nbs^2^* | 92 | 65 (71) | 20 (22) |
| *nbs^2^ / Df(3L)AC1* | 137 | 90 (66) | 33 (24) |
| *nbs^1^ / nbs^2^* | 173 | 128 (74) | 11 (6.4) |
| *nbs^1^,P[nbs^+^] / Df(3L)AC1* | 195 | 2 (1.0) | 0 |

^a^ The percent anaphases with bridges is shown in parenthesis.

^b^ Number of anaphases with a lagging chromosome fragment. The percent of anaphases with a lagging chromosome fragment is shown in parenthesis.
